# Thermoelectric properties of armchair graphene nanoribbons with array characteristics

**DOI:** 10.1039/d3ra07863a

**Published:** 2024-01-22

**Authors:** David M. T. Kuo

**Affiliations:** a Department of Electrical Engineering and Department of Physics, National Central University Chungli 32001 Taiwan mtkuo@ee.ncu.edu.tw

## Abstract

The thermoelectric properties of armchair graphene nanoribbons (AGNRs) with array characteristics are investigated theoretically using the tight-binding model and Green's function technique. The AGNR structures with array characteristics are created by embedding a narrow boron nitride nanoribbon (BNNR) into a wider AGNR, resulting in two narrow AGNRs. This system is denoted as *w*-AGNR/*n*-BNNR, where ‘*w*’ and ‘*n*’ represent the widths of the wider AGNR and narrow BNNR, respectively. We elucidate the coupling effect between two narrow symmetrical AGNRs on the electronic structure of *w*-AGNR/*i*-BNNR. A notable discovery is that the power factor of the 15-AGNR/5-BNNR with the minimum width surpasses the quantum limitation of power factor for 1D ideal systems. The energy level degeneracy observed in the first subbands of *w*-AGNR/*n*-BNNR structures proves to be highly advantageous in enhancing the electrical power outputs of graphene nanoribbon devices.

## Abstract 2

The thermoelectric properties of armchair graphene nanoribbons (AGNRs) with array characteristics are investigated theoretically using the tight-binding model and Green's function technique.

## Graphical abstract

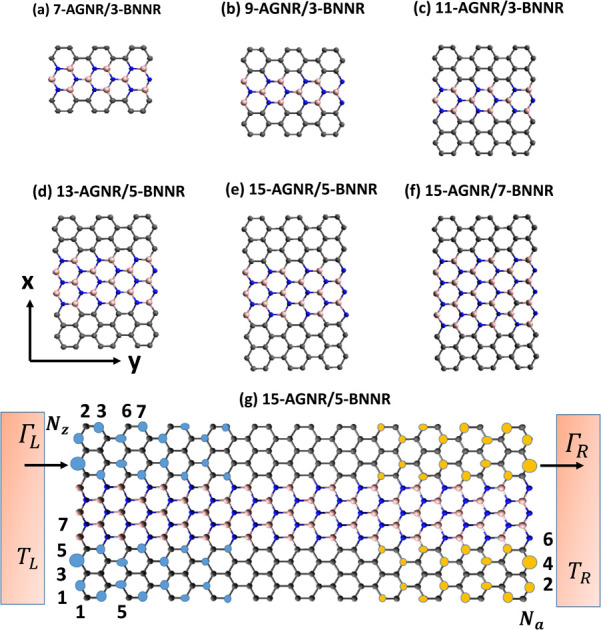

## 1 Introduction

Extensive research efforts have been dedicated to exploring the potential applications of graphene nanoribbons (GNRs) across various fields such as electronics, optoelectronics, and thermoelectric devices. This interest has surged since the groundbreaking discovery of two-dimensional graphene in 2004 by Novoselov and Geim.^1^ Despite significant strides, GNR-based devices face a pronounced challenge in amplifying their electrical and optical power outputs. The limited power outputs are attributed to the low transmission coefficient in the band edges of the first subbands of GNRs.^2–11^ In the context of GNR-based device applications, the electronic states proximate to the band edges of the initial conduction and valence subbands play a pivotal role in optical and transport processes. Consequently, it becomes imperative to engineer band-edge electronic states with a high transmission coefficient, paving the way for the development of electronic and thermoelectric devices capable of enhancing their electrical power outputs.

Different classes of GNRs have undergone thorough theoretical and experimental investigations by diverse research groups. These encompass armchair GNRs (AGNRs),^12,13^ zigzag GNRs (ZGNRs),^14^ cove-edged zigzag GNRs (CZGNRs),^15–18^ AGNR heterojunctions,^19–24^ and graphene quantum dot superlattices.^25,26^ Typically, these quasi-one-dimensional systems exhibit suboptimal transmission coefficients near the edge states of the first conduction and valence subbands when coupled with electrodes.^27–32^ In the realm of ballistic transport, under ideal conditions and neglecting electron spin degeneracy, the one-dimensional transmission coefficient for electrons in the first conduction and valence subbands is anticipated to be unity. However, defects and contact effects in finite GNRs inevitably introduce backward scattering, reducing the transmission coefficient to less than one for specific electron wavelengths near the band edge of the first subbands.^27–32^ Additionally, the contact geometries between the graphene electrodes and the molecules play a significant role in influencing the transmission coefficient of electron transport in the molecules.^33–36^ Consequently, achieving an energy-dependent transmission coefficient of one for electrons across all wavelengths becomes challenging when finite GNRs are connected to electrodes. It was demonstrated that in the case of quantum dot (QD) molecules with high orbital degeneracy, a greater degree of degeneracy leads to a higher transmission coefficient. This increase in transmission coefficient enhances electrical conductance while keeping the Seebeck coefficient unchanged. Consequently, such enhancement results in increased electrical power outputs in QD-based thermoelectric devices.^37^

**Fig. 1:**
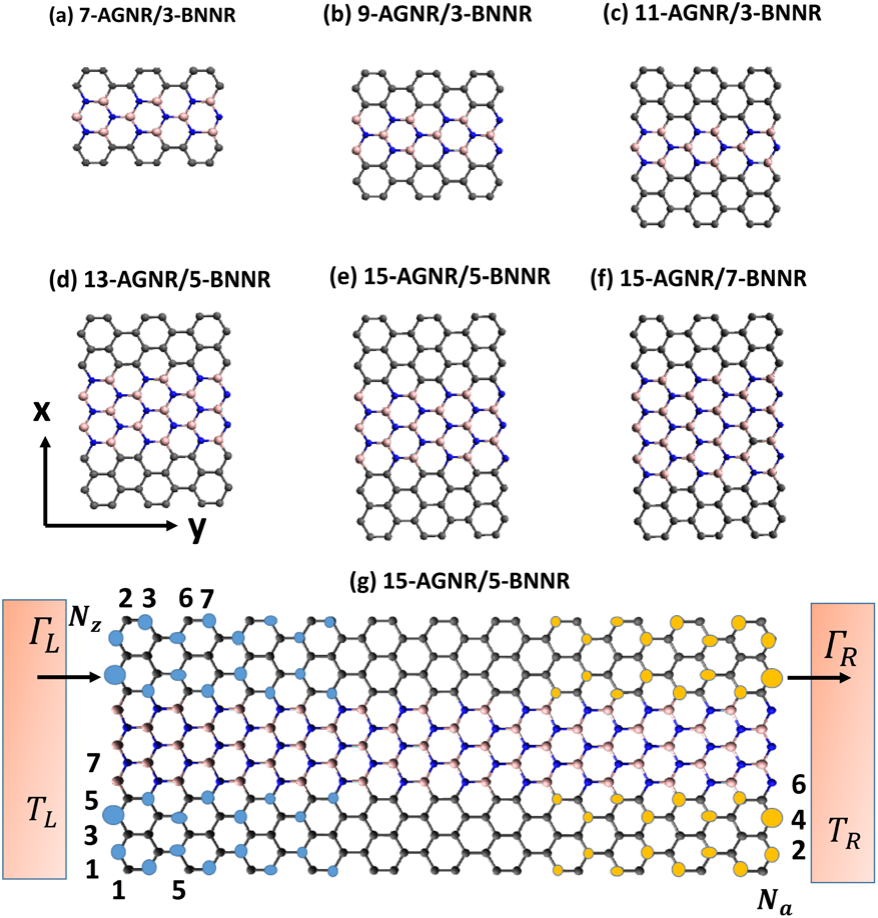
Schematic diagram depicts hybridized *w*-AGNR/*n*-BNNR structures, where the notations *w* and *n* refer to the wide and narrow widths, respectively. Panels (a)–(f) showcase six different scenarios, while panel (g) illustrates the line-contacting of zigzag-edge atoms in a 15-AGNR/5-BNNR structure to electrodes. Symbols *Γ*_L_ (*Γ*_R_) represent the electron tunneling rate between the left (right) electrode and the leftmost (rightmost) atoms at the zigzag edges, and *T*_L_ (*T*_R_) denotes the equilibrium temperature of the left (right) electrode. The charge densities of *ε*_e,1_ = 0.3741 eV and *ε*_h,1_ = −0.3932 eV for 15-AGNR/5-BNNR structure are depicted in panel (g), with light-blue and orange circles representing the charge densities for *ε*_e,1_ and *ε*_h,1_, respectively. The radius of the circle represents the intensity of the charge density.

In this study we propose an innovative configuration wherein a narrow boron nitride nanoribbon (BNNR) is seamlessly integrated into a wider AGNR. The realization of such a structure can be achieved through advanced DUV lithographic techniques.^38^ We explore AGNRs and BNNRs with varying widths, spanning from 7 to 19 for AGNRs and 3 to 9 for BNNRs. These configurations are denoted as *w*-AGNR/*n*-BNNR, illustrating scenarios where electrons transport along the armchair direction. Fig. 1(a)–(f) portray six instances of *w*-AGNR/*n*-BNNR structures, which can alternatively be interpreted as *u*-AGNR/*m*-BNNR/*b*-AGNR heterostructures, where *u*, *m*, and *b* represent the widths of the upper AGNR, middle BNNR, and bottom AGNR, respectively. A noteworthy discovery from our study is that the power factor of the 15-AGNR/5-BNNR, forming a two narrow symmetrical AGNR array, surpasses the quantum limitation of the power factor for one-dimensional ideal systems. The orbital degeneracy in the first subbands of 15-AGNR/5-BNNR, characterized by a substantial band gap, significantly enhances electrical conductance. Meanwhile, the semiconducting phase of two narrow AGNRs maintains an unchanged Seebeck coefficient. Consequently, the power factor of the 15-AGNR/5-BNNR junction is greatly enhanced.

## 2 Calculation methodology

To explore the thermoelectric properties of *w*-AGNR/*n*-BNNR connected to the electrodes, we utilize a combination of the tight-binding model and the Green's function technique. The system Hamiltonian consists of two components: *H* = *H*_0_ + *H*_GNR_. Here, *H*_0_ signifies the Hamiltonian of the electrodes, encompassing the interaction between the electrodes and the *w*-AGNR/*n*-BNNR. Meanwhile, *H*_GNR_ represents the Hamiltonian for the *w*-AGNR/*n*-BNNR and can be expressed as follows:1
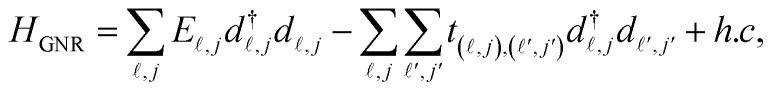


Here, *E*_*ℓ*,*j*_ represents the on-site energy of the orbital in the *ℓ*-th row and *j*-th column. The operators *d*^†^_*ℓ*,*j*_ and *d*_*ℓ*,*j*_ create and annihilate an electron at the atom site denoted by (*ℓ*, *j*). The parameter *t*_(*ℓ*,*j*)(*ℓ*′,*j*′)_ characterizes the electron hopping energy from site (*ℓ*′, *j*′) to site (*ℓ*, *j*). We assign the tight-binding parameters for *w*-AGNR/*n*-BNNR as follows: *E*_B_ = 2.329 eV, *E*_N_ = −2.499 eV, and *E*_C_ = 0 eV to boron, nitride, and carbon atoms, respectively. To simplify our analysis, we have neglected variations in electron hopping strengths between different atoms due to their relatively minor differences.^39^ We set *t*_(*ℓ*,*j*)(*ℓ*′,*j*′)_ = *t*_ppπ_ = 2.7 eV for the nearest-neighbor hopping strength. We can utilize these parameters to replicate the bandgaps of the BNNR/AGNR/BNNR structure as illustrated in Fig. 3(d) of ref. 40, which were originally calculated using the first-principle method.

In the linear response region, the electrical conductance (*G*_e_), Seebeck coefficient (*S*) and electron thermal conductance (*κ*_e_) can be computed using 
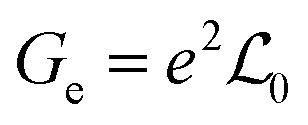
, 
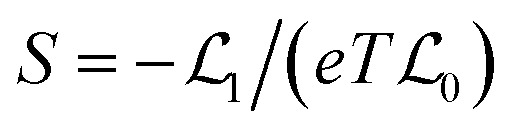
 and 
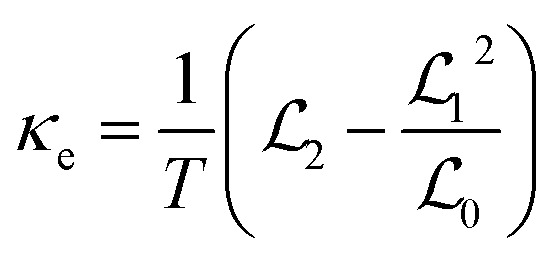
 with 
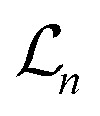
 (*n* = 0, 1, 2) defined as2
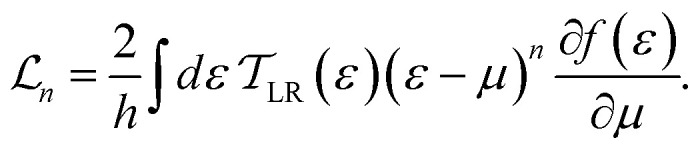


Here, *f*(*ε*) = 1/(1 + exp((*ε* − *μ*)/*k*_B_*T*)) represents the Fermi distribution function of electrodes at equilibrium chemical potential *μ*. The constants *e*, *h*, *k*_B_, and *T* denote the electron charge, Planck's constant, Boltzmann's constant, and the equilibrium temperature of the electrodes, respectively. 
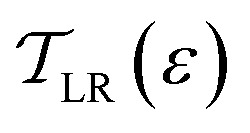
 signifies the transmission coefficient of a *w*-AGNR/*n*-BNNR connected to electrodes, and it can be calculated using the formula 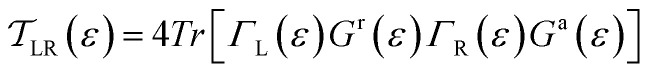,^32^ where *Γ*_L_(*ε*) and *Γ*_R_(*ε*) denote the tunneling rate (in energy units) at the left and right leads, respectively, and *G*^r^(*ε*) and *G*^a^(*ε*) are the retarded and advanced Green's functions of the GNRs, respectively. The tunneling rates are determined by the imaginary part of the self-energy originating from the coupling between the left (right) electrode and its adjacent GNR atoms. In terms of tight-binding orbitals, *Γ*_α_(*ε*) and Green's functions are matrices. For simplicity, *Γ*_α_(*ε*) for interface atoms possesses diagonal entries with a common value of *Γ*_t_.^32^ When graphene is connected to metal electrodes, contact properties such as the Schottky barrier or ohmic contact can exert a substantial impact on electron transport in graphene.^31^ Despite numerous theoretical studies striving to elucidate this crucial behavior from first principles, the theoretical limitations result in obtaining only qualitative results regarding *Γ*_t_ arising from the contact junction.^41^ The thermoelectric figure of merit is calculated by *ZT* = *S*^2^*G*_e_*T*/(*κ*_e_ + *κ*_ph_), where *κ*_ph_ is the phonon thermal conductance of GNRs.

## 3 Results and discussion

### 3.1 Electronic structures of *w*-AGNR/*n*-BNNR structures

**Fig. 2:**
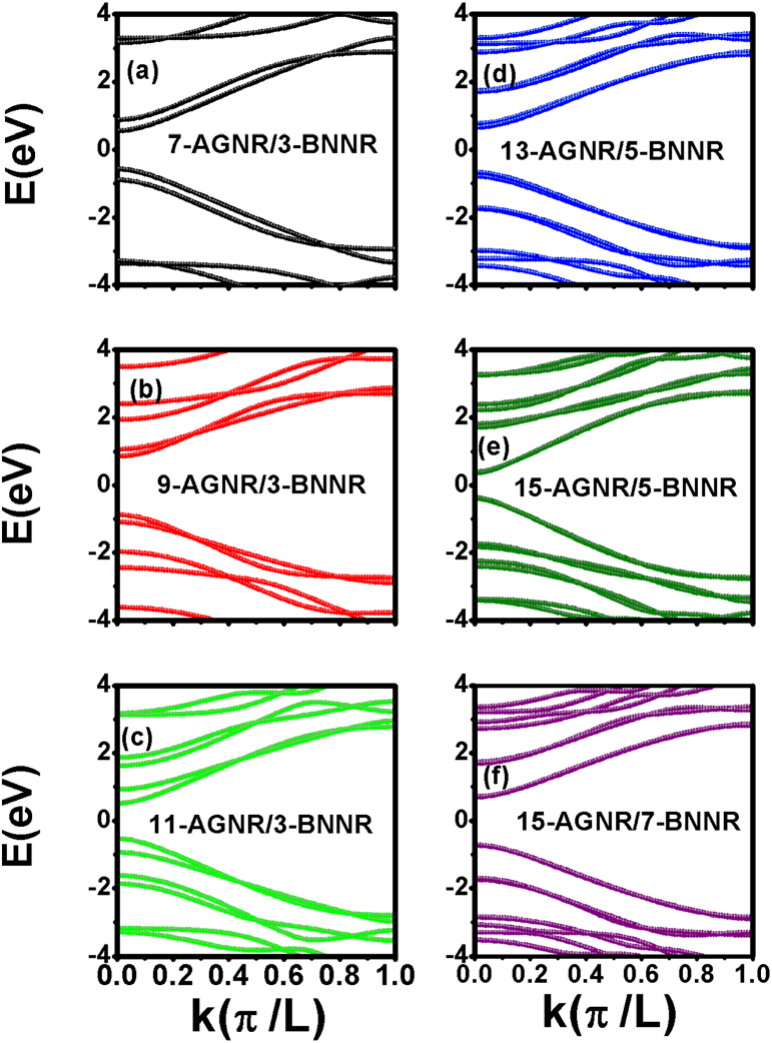
Electronic subband structures of various *w*-AGNR/*n*-BNNR structures: (a) 7-AGNR/3-BNNR, (b) 9-AGNR/3-BNNR, (c) 11-AGNR/3-BNNR, (d) 13-AGNR/5-BNNR, (e) 15-AGNR/5-BNNR, and (f) 15-AGNR/7-BNNR.

The electronic behavior of AGNRs is primarily determined by their widths, which adhere to the rule *N*_z_ = 3*p*, *N*_z_ = 3*p* + 1, and *N*_z_ = 3*p* + 2, where *p* is an integer. Specifically, AGNRs exhibit semiconducting behavior for *N*_z_ = 3*p* and *N*_z_ = 3*p* + 1, while AGNRs with *N*_z_ = 3*p* + 2 exhibit either metallic behavior or possess small band gaps in their electronic structures.^42^ To illustrate the impact of BNNRs, the electronic structures of various *w*-AGNR/*n*-BNNR structures (*u*-AGNR/*m*-BNNR/*b*-AGNR heterostructures) are presented in Fig. 2(a)–(f).

As depicted in Fig. 2(a)–(f), *w*-AGNR/*n*-BNNR structures distinctly exhibit semiconducting phases. However, for widths *w* = 7, 9, and 11, we observe the absence of degeneracy in the first subbands, attributed to the narrow barrier width between *u*-AGNR and *b*-AGNR, which arises from BNNR with *n* = 3. Insufficient width of 3-BNNR indicates coupling between two narrow symmetrical AGNRs. This coupling effect induces bonding and antibonding energy levels, lifting orbital degeneracy. The lack of orbital degeneracy indicates a *w*-AGNR/3-BNNR without the characteristic of an AGNR array. On the other hand, the degeneracy of the first subbands exists in the case of the 15-AGNR/5-BNNR and 15-AGNR/7-BNNR structures. In these situations, *w*-AGNR/*n*-BNNR structures exhibit AGNR array characteristics. It is worth noting that the 5-AGNRs within the 15-AGNR/5-BNNR structure exhibit semiconducting phases with a band gap of *E*_gap_ = 0.7 eV, contradicting the characteristic metallic phase associated with AGNRs of width *N*_z_ = 5.^14^

The original small band gaps of 5-AGNRs are enlarged due to a change in one of the boundary conditions, transitioning from the vacuum potential barrier to the potential barrier of BNNR. This outcome aligns with the results predicted by the first principle method,^40^ where the authors considered 5-AGNRs confined by two BNNRs. By artificially setting the energy levels of nitride and boron atoms to a large value, resembling a vacancy, the 5-AGNRs revert to metallic phases.

### 3.2 Finite *w*-AGNR/*n*-BNNR structures

**Fig. 3:**
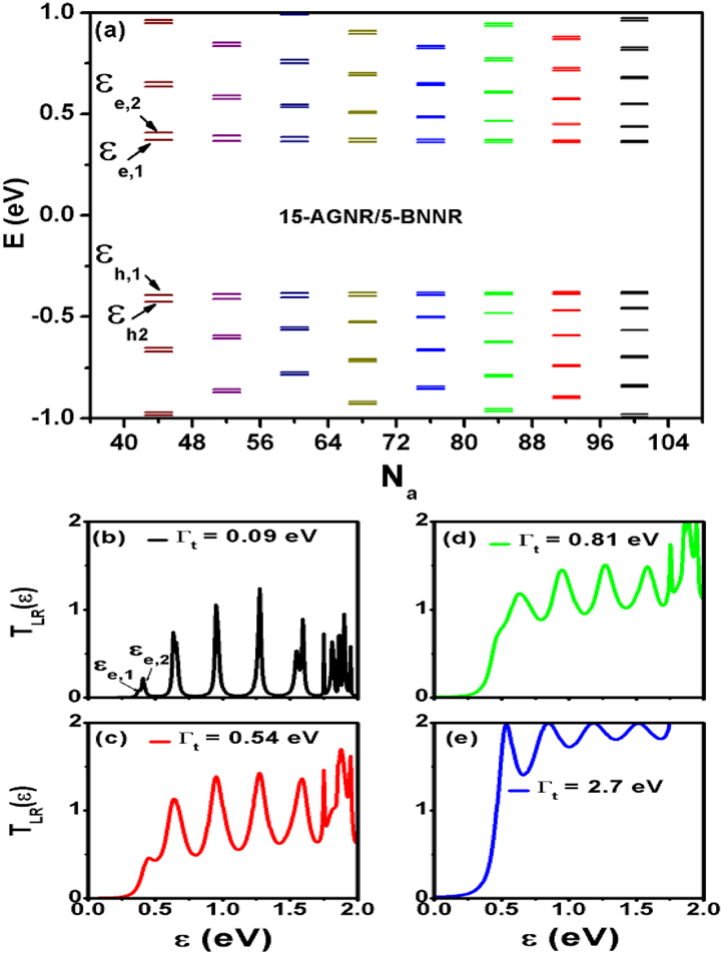
Energy levels of finite 15-AGNR/5-BNNR structures for various *N*_a_ values (a). Transmission coefficient 
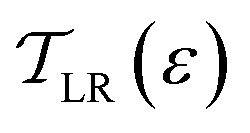
 of 15-AGNR/5-BNNR with *N*_a_ = 44 (*L*_a_ = 4.54 nm) for different *Γ*_t_ values. (b) *Γ*_t_ = 0.09 eV, (c) *Γ*_t_ = 0.54 eV, (d) *Γ*_t_ = 0.81 eV, and (e) *Γ*_t_ = 2.7 eV.

In the fabrication of GNR-based devices, ensuring that the channel length is smaller than the electron mean free path is a crucial requirement for achieving ballistic transport. To explore the influence of channel length and contacts, we compute the energy levels of 15-AGNR/5-BNNR structures that are decoupled from the electrodes. The calculated results are presented in Fig. 3(a). The energy levels, labeled as *ε*_e,1_ = 0.3741 eV and *ε*_h,1_ = −0.3932 eV, are found to be relatively insensitive to variations in *N*_a_ within the range of 44 to 100. However, the energy level separation *Δ*_e_ (*Δ*_h_) between *ε*_e,1_ (*ε*_h,1_) and *ε*_e,2_ (*ε*_h,2_) diminishes as *N*_a_ is increased.

The charge densities for *ε*_e,1_ = 0.3741 eV and *ε*_h,1_ = −0.3932 eV are depicted in Fig. 1(g), with light-blue and orange circles representing the charge densities for *ε*_e,1_ and *ε*_h,1_, respectively. These charge densities exhibit a decay along the armchair directions. Based on the distribution of charge density, it becomes evident that *ε*_e,1_ and *ε*_h,1_ correspond to the end zigzag edge states of finite AGNRs. The presence of BNNRs causes these energy levels of end zigzag edge states in AGNRs to shift from zero energy modes to *ε*_e,1_ (*ε*_h,1_). For energy values within the range 0 < *ε* < 1 eV, there are six energy levels in the case of *N*_a_ = 44. As depicted in Fig. 3(b)–(e), the calculated transmission coefficient 
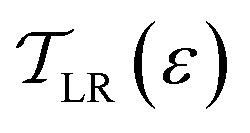
 for the 15-AGNR/5-BNNR structure with *N*_a_ = 44 (*L*_a_ = 4.54 nm) clearly reveals these six energy levels only for a small tunneling rate *Γ*_t_ = 0.09 eV.

In the case of a large tunneling rate, *Γ*_t_ = 2.7 eV, as depicted in Fig. 3(e), which can be considered as resembling graphene electrodes, the maximum values of 
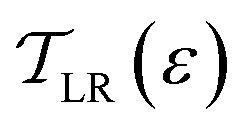
 reach two in the first conduction subband. This can be regarded as evidence showcasing 15-AGNR/5-BNNR with AGNR array characteristic. It is important to note that the area under the 
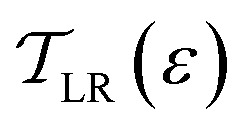
 curve for *Γ*_t_ = 2.7 eV is maximized. Henceforth, we focus on the case with *Γ*_t_ = 2.7 eV throughout this article.

**Fig. 4:**
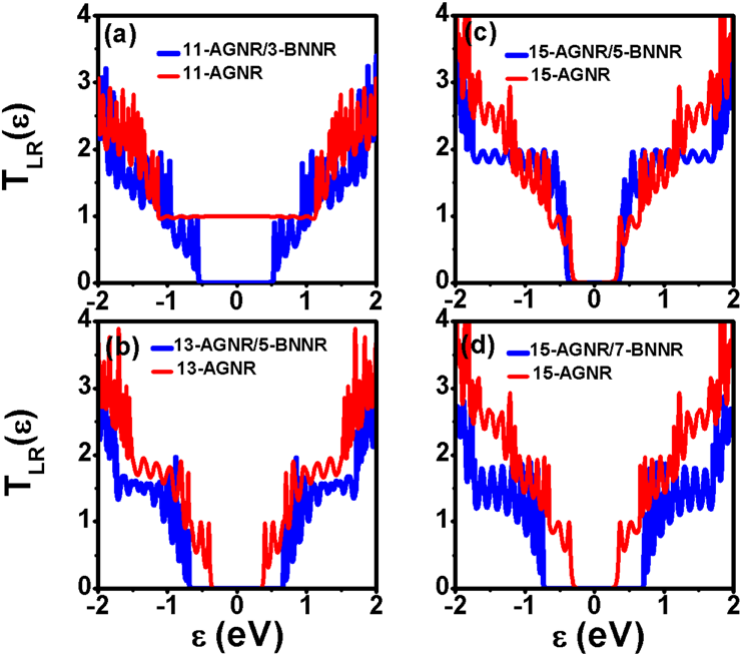
Transmission coefficients 
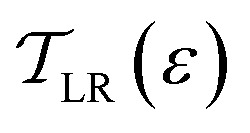
 of *w*-AGNR/*n*-BNNR structures and *w*-AGNR structures with *N*_a_ = 84 (*L*_a_ = 8.8 nm) and *Γ*_t_ = 2.7 eV. (a) 11-AGNR/3-BNNR and 11-AGNR, (b) 13-AGNR/5-BNNR and 13-AGNR, (c) 15-AGNR/5-BNNR and 1D ideal case, and (d) 15-AGNR/7-BNNR and 15-AGNR.

To investigate the impact of BNNRs on the transmission coefficients of AGNR structures, we present the calculated 
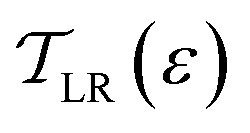
 for four different *w*-AGNR/*n*-BNNR structures, all characterized by *N*_a_ = 84 (*L*_a_ = 8.8 nm) and *Γ*_t_ = 2.7 eV in Fig. 4. In Fig. 4(a), we observe that the 11-AGNR transitions from a metallic phase to a semiconducting phase when 3-BNNR is embedded within it. However, the maximum values of 
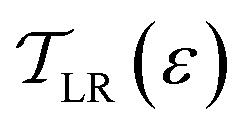
 in the first conduction and valence subbands only reach one. As the second subbands emerge around *ε* ≈ 1 eV, 
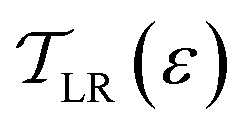
 can attain a value of two. This behavior can be understood by referencing the electronic structure in Fig. 2(c).

In Fig. 4(b), we observe that the band gap of the semiconducting 13-AGNR is widened when 5-BNNR is integrated into the 13-AGNR structure. Moreover, its maximum 
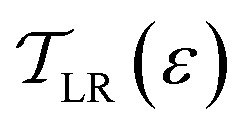
 can reach two within the first subbands. However, the occurrence of 
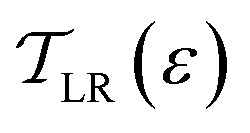
 equal to two is confined to specific energy ranges. Fig. 4(c) demonstrates that the transmission coefficient curve of the 15-AGNR/5-BNNR exhibits a larger area within the first conduction and valence subbands compared to the 1D-ideal case with a rectangular shape. Conversely, in the case of 7-BNNR, as shown in Fig. 4(d), the area of 
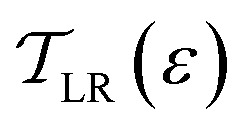
 is reduced when compared to the 5-BNNR scenario. It's worth noting that the 15-AGNR/7-BNNR, corresponding to the 4-AGNR/7-BNNR/4-AGNR heterostructure, contains two 4-AGNRs. The transmission coefficient of *u*-AGNR/*m*-BNNR/*b*-AGNR structures favors *u*-AGNR and *b*-AGNR with *N*_z_ = 3*p* + 2 widths. For wider AGNR structures like 17-AGNR and 19-AGNR, such as 17-AGNR/7-BNNR and 19-AGNR/9-BNNR, their transmission coefficients yield results similar to those of the 15-AGNR/5-BNNR. If we consider the narrowest width in *w*-AGNR/*n*-BNNR structures, it becomes evident that the 15-AGNR/5-BNNR structure will exhibit the highest power factor.

### 3.3 Thermoelectric properties of finite 15-AGNR/5-BNNR structures

**Fig. 5:**
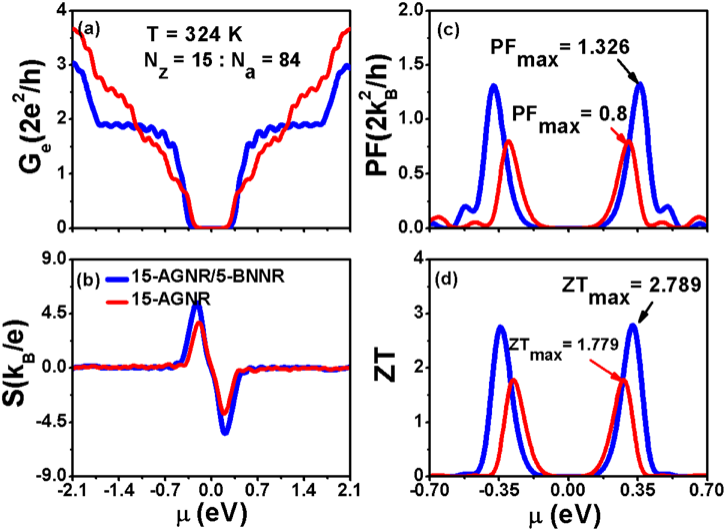
(a) Electrical conductance (*G*_e_), (b) Seebeck coefficient (*S*), (c) power factor (PF = *S*^2^*G*_e_), and (d) figure of merit of 15-AGNR/5-BNNR and 15-AGNR structures with a ribbon length of *N*_a_ = 84 (*L*_a_ = 8.8 nm), as functions of chemical potential (*μ*) at a temperature of 324 K.

In this subsection, we present the calculated electrical conductance (*G*_e_), Seebeck coefficient (*S*), power factor (PF = *S*^2^*G*_e_), and figure of merit (*ZT*) for both 15-AGNR/5-BNNR and 15-AGNR structures as functions of chemical potential at a temperature of 324 K and a nanoribbon width of *N*_a_ = 84 (*L*_a_ = 8.8 nm), as depicted in Fig. 5. We use specific constants for the units: *G*_0_ = 2*e*^2^/*h* = 77.5 μS for electrical conductance, *k*_B_/*e* = 86.25 μV K^−1^ for the Seebeck coefficient, and 2*k*^2^_B_/*h* = 0.575 pW K^−2^ for the power factor. In Fig. 5(a), we observe that the electrical conductance of the first conduction subband exhibits a two-fold quantum conductance value for the 15-AGNR/5-BNNR structure. This significant enhancement in *G*_e_ can be attributed to the AGNR array characteristic. In Fig. 5(b), the Seebeck coefficient is also enhanced due to the large band gap. The combined enhancement of *G*_e_ and *S* results in a substantial maximum power factor value of PF = 1.326 in Fig. 5(c). It's worth noting that the maximum power factors of AGNRs with *N*_a_ = 84 for various ribbon widths (*N*_z_ = 7, *N*_z_ = 9, *N*_z_ = 13, and *N*_z_ = 15) are as follows: 0.4457, 0.7057, 0.667, and 0.803, respectively. The maximum power factor of the 15-AGNR/5-BNNR structure not only surpasses these maximum PF values but also exceeds the theoretical limit, PF_QB_ = 1.2659, as established for one-dimensional ideal systems by Whitney.^43^

The thermoelectric figure of merit, denoted as *ZT*, is determined by the formula 
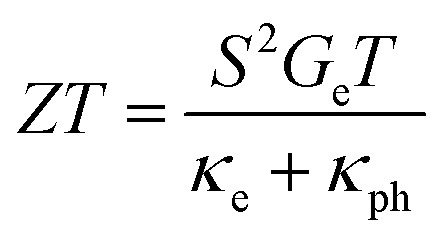
, where *κ*_ph_ represents the phonon thermal conductance of 15-AGNR/5-BNNR structure. For simplicity, we consider *κ*_ph_ = *F*_s_ × *κ*_GNR_. Here, 
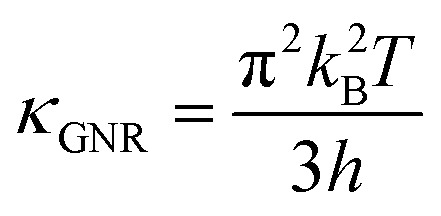
 denotes the phonon quantum conductance of 15-AGNRs. *F*_s_ = 0.1 denotes a reduction factor resulting from *w*-AGNR/*n*-BNNR heterostructures. It has been theoretically demonstrated that the magnitude of *κ*_ph_ can be reduced by one order magnitude for AGNRs with a BN interface.^44^ The calculated *ZT* values for 15-AGNR/5-BNNR and 15-AGNR structures are presented in Fig. 5(d). Due to the enhanced power factor, the *ZT* of 15-AGNR/5-BNNR is increased by 56% compared to that of 15-AGNR, which exhibits *ZT* = 1.779. It should be noted that in the calculations of *ZT* values, the phonon thermal conductance for 15-AGNR/5-BNNR and 15-AGNR structures is assumed to be the same. However, it is expected that the *κ*_ph_ of 15-AGNR/5-BNNR is smaller than that of 15-AGNR.^44^ Consequently, the 56% enhancement in *ZT* for 15-AGNR/5-BNNR may be considered a conservative estimate.

## 4 Conclusion

In our study, we investigated the electron transport properties along the armchair edge direction of *w*-AGNR/*n*-BNNR. Notably, when the zigzag edge atoms of these heterostructures make direct contact with the electrodes, the transmission coefficient area of the 5-AGNR/5-BNNR/5-AGNR structure surpasses the ideal 1D case, as illustrated in Fig. 4(c). This remarkable outcome can be attributed to two crucial factors: (a) The electronic properties of 5-AGNRs undergo a transition from metallic phases to semiconducting phases when the BNNR potential barrier height replaces one of the two vacuum potential barrier heights. (b) The 15-AGNR/5-BNNR structure exhibits AGNR array characteristics. The formal factor leaves the Seebeck coefficient unchanged, and the latter factor significantly enhances the electrical conductance. The maximum power factor achieved by the finite 5-AGNR/5-BNNR/5-AGNR structure, with a ribbon length of *N*_a_ = 84 (*L*_a_ = 8.8 nm), reaches 
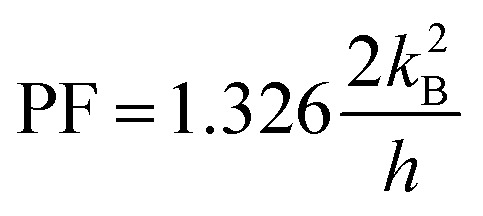
 at a temperature of 324 K, surpassing the quantum limit for the power factor in 1D systems, 
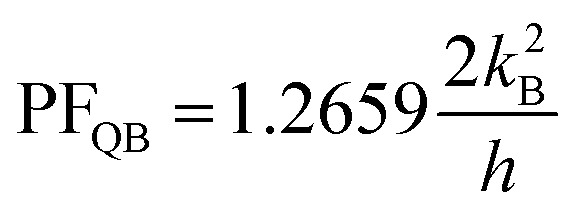
. Due to the mechanical strength and flexibility of GNR thermoelectric generators (TEGs), the 15-AGNR/5-BNNR structures show promise for utilizing semiconducting GNR-based TEGs in various applications, including wearable electronics.^45^ Our design not only represents a significant advancement in GNR-based thermoelectric devices but also holds the potential for enhancing the performance of GNR-based optoelectronics.^11^

## Conflicts of interest

There are no conflicts to declare.

## Supplementary Material
